# Collagenous colitis following SARS‐Cov2 mRNA vaccination

**DOI:** 10.1002/jgh3.12885

**Published:** 2023-03-10

**Authors:** Momoko Iketani, Yutaka Takada, Toshinao Itani

**Affiliations:** ^1^ Department of Gastroenterology and Hepatology Kobe City Nishi‐Kobe Medical Center Kobe Japan

**Keywords:** collagenous colitis, microscopic colitis, SARS‐Cov2 mRNA vaccination

## Abstract

A healthy 49‐year‐old female developed intractable watery diarrhea after the Pfizer SARS‐Cov2 mRNA vaccination. She was subsequently diagnosed with collagenous colitis (CC). She had no prior history of medication use, suggesting of vaccination being the trigger. CC or lymphocytic colitis should be considered as differential diagnoses for persistent watery diarrhea after SARS‐Cov2 mRNA vaccination.

## Graphical abstract

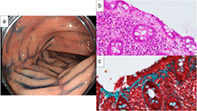

## Introduction

Collagenous colitis (CC) and lymphocytic colitis (LC) are inflammatory bowel diseases characterized by chronic, watery, non‐bloody diarrhea. Several drugs and other autoimmune diseases have been associated with CC and LC. In addition to this, these days, a few cases of CC or LC have been reported as adverse events of SARS‐Cov2 mRNA vaccination. Here we report a case of CC occurring after the Pfizer SARS‐Cov2 mRNA vaccination.

## Case report

**Figure 1:**
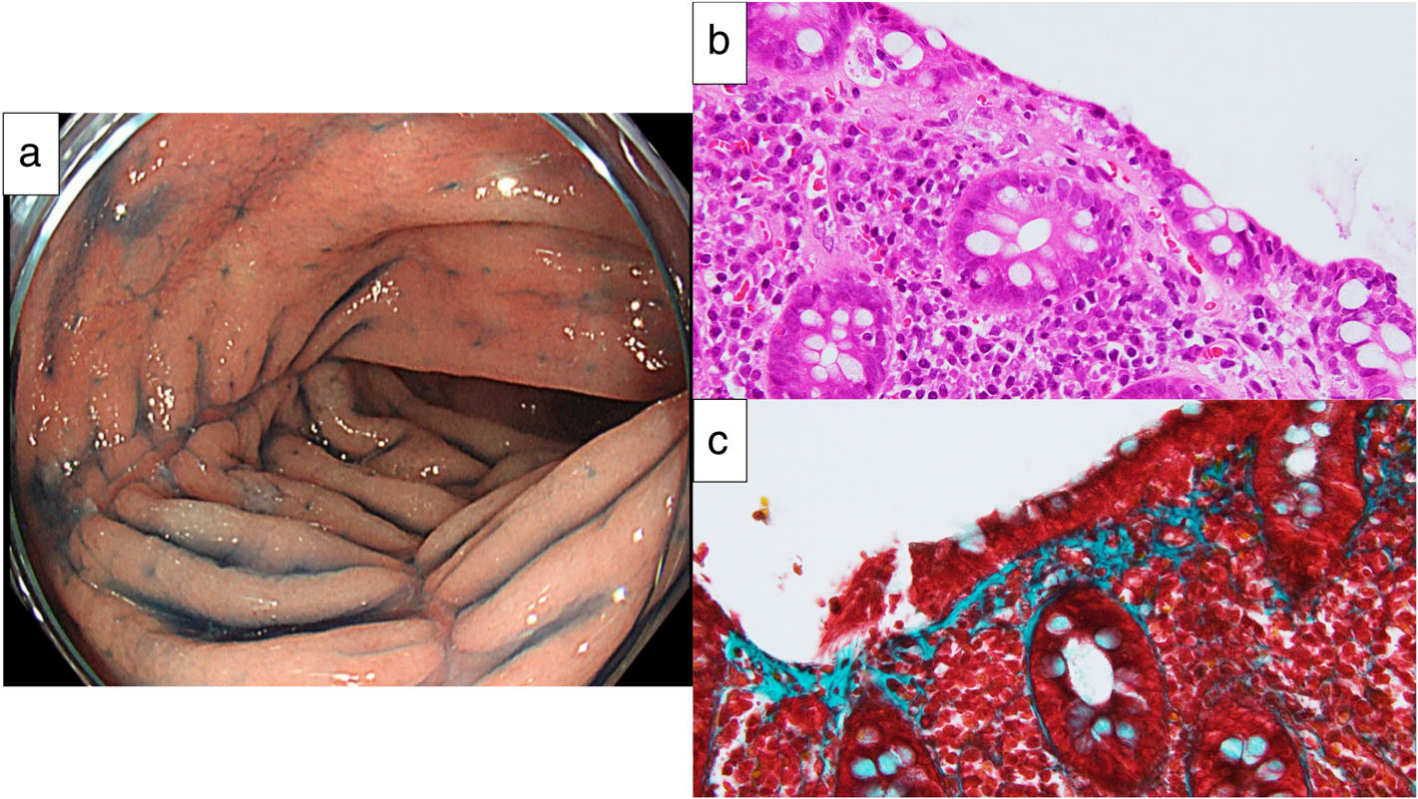
(a) Colonoscopy showed ulcer scars running longitudinally across the sigmoid colon. (b) Pathological examination (H&E) showed infiltration of lymphocytes and plasma cells into the lamia propria, and a subepithelial collagen band over 10 μm. (c) Masson's trichrome staining showed the subepithelial collagen band more clearly.

A 49‐year‐old otherwise healthy woman presented with intractable watery diarrhea (10–20 times/day) without abdominal pain or bloody stools. The symptoms began 3 days after her first Pfizer SARS‐Cov2 mRNA vaccination, but improved after 2 months. However, 3 days after her third vaccination, watery diarrhea flared up; antidiarrheal medications did not work. Despite frequent watery diarrhea, the patient's general condition was relatively good, with no abdominal pain, fever, arthritis, or skin rash. A stool culture test was negative, and a blood test showed no obvious abnormalities including HbA1c, thyroid function, IgA, IgG, IgM, and antinuclear antibodies. Colonoscopy showed ulcer scars running longitudinally across the sigmoid colon (Fig. 1a), and CC was diagnosed by biopsy, which showed a thickened subepithelial collagen band (Fig. 1b,c). Ninety days later, the diarrhea had spontaneously resolved.

## Discussion

CC is a subtype of microscopic colitis (MC), in addition to LC.^1^ CC is often caused by drugs such as antiacids and nonsteroidal anti‐inflammatories.^2^ An association with inflammatory or autoimmune diseases such as rheumatoid arthritis, thyroid disorders, coeliac disease, and diabetes mellitus have also been suggested.^3^ A characteristic endoscopic finding of CC is longitudinal ulcers called mucosal tears. The longitudinal ulcers with well‐defined borders and lack of surrounding edema and erythema are different from those of ulcerative colitis and Crohn's disease. The definitive diagnosis is based on histopathological examination, which shows thickening of the collagen band just below the epithelium to more than 10 μm and inflammatory cell infiltration in the submucosa.^4^ For management, patients should be advised to avoid medications associated with MC and use antidiarrheals. In active cases, oral budesonide may be effective for achieving induction and maintenance of both clinical and histological response.^5^ Our case had no prior history of medication use other than antidiarrheals, and there were no symptoms or laboratory findings suspicious of other inflammatory or autoimmune diseases. Symptoms occurred a few days after vaccination and resolved within 2 months, suggesting of vaccination being the trigger. The Centers for Disease Control and Prevention's vaccine adverse event database shows four cases of CC and five cases of LC following SARS‐Cov2 mRNA vaccination. Therefore, CC or LC should be considered as differential diagnoses for persistent watery diarrhea after SARS‐Cov2 mRNA vaccination.

### Patient consent statement

Informed consent was obtained from the patient for publication of this case report and accompanying images.
